# A novel method to investigate the effects of gene mutations at the cellular level using a dual expression lentiviral vector

**DOI:** 10.1042/BSR20182383

**Published:** 2019-05-02

**Authors:** Liyun Huang, Feixia Peng, Yun Wei, Wei He, Shasha Zhao, Juan Wang, Yang Zhang, Houliang Zhao, Wensheng Deng

**Affiliations:** College of Life Science and Health, Wuhan University of Science and Technology, Wuhan 430065, China

**Keywords:** cellular level, dual expression vector, gene mutation effect, gene transcription, method

## Abstract

One of the conventional methods to study the effects of gene mutations is that gene mutants are transfected into mammalian cells, and the dominant effects of gene mutants in the cells are examined. However, the result obtained using this method is not always satisfactory due to the interference of endogenous expression. Whether there is a better method to investigate the effects of gene mutations in cells remains to be examined. In the present study, a novel dual expression lentiviral vector was constructed using a shRNA-expressing lentiviral vector and combined techniques. Using this dual expression system, the vectors expressing both transcription factor IIA γ (TFIIAγ) shRNA and HA-TFIIAγ or its mutants were generated, and the effects of *TFIIAγ* gene mutations on transcription and protein–DNA interaction were investigated. We show that the transfection of the vector expressing TFIIAγ shRNA and *HA-TFIIAγ* fusion gene was able to silence the expression of endogenous *TFIIAγ* gene but not affect that of exogenous *HA-TFIIAγ* fusion gene in either transiently transfected cells or stable cell lines. Mutations in the conservative domain between AA_62_ and AA_69_ in TFIIAγ inhibit the activities of promoters and endogenous gene expression, and reduce TFIIAγ binding to AdML core promoter compared with wild-type (WT) TFIIAγ. ChIP-qPCR data suggest that the TFIIAγ N63A mutant inhibits insulin-like growth factor 2 (IGF2) transcription by reducing the recruitments of TFIIAγ, polymerase II (Pol II), TATA box-binding protein (TBP), and TBP associated factor 1 (250 kDa) (TAF1) at its promoter. Our study provides a novel method that is used to investigate the effects of gene mutations at the cellular level.

## Introduction

Site-directed mutagenesis is one of the most important experimental techniques in molecular biology and is widely used to investigate the structure and function of DNA and protein molecules, the interaction between proteins, organ development, and others [1–5]. Gene mutations can be achieved in the laboratories using various approaches [6–9]; among which, PCR site-directed mutagenesis and genome editing techniques are most frequently used [8,10,11]. The PCR site-directed mutagenesis is mainly used for generation of gene mutants *in vitro* [8]; whereas, the genome editing techniques – including the methods mediated by ZFNs, TALEN, and CRISPR-Case9 – are powerful tools to generate mutations in the genome of a living organism [12–14]. However, off-target mutagenesis has been described where gene mutations are generated using genome editing approaches [15].

Previous studies have shown that the PCR site-directed mutagenesis can be used to map the interacting sites between two proteins or between protein and DNA *in vitro* [16–18], and that the gene mutants obtained by this approach can also be transfected into cells, the dominant effects of gene mutants are subsequently analyzed [19,20]. However, the result obtained with this method is not always satisfactory due to the interference of endogenous gene expression. To exclude the interference from endogenous gene expression, another approach has been developed in the late studies where endogenous gene expression in cells was silenced by siRNA prior to transfection of an exogenous wild-type (WT) gene or its mutants [21,22]. However, the introductions of siRNA-suppressing endogenous expression and mutated gene separately are not only costly but also time-consuming. To overcome these drawbacks, it is important to develop a more effective method to study the effects of gene mutations.

Lentiviral expression vector has widely been used in basic research, gene therapy, and vaccine production [23–25]. Previous study has shown that both shRNA and protein can be simultaneously expressed using a dual expression lentiviral vector [26]; suggesting that the dual expression lentiviral vector could be applied to investigating the effects of gene mutations in cells. In the present study, a novel dual expression lentiviral vector was constructed using a shRNA-expressing lentiviral vector and combined DNA manipulation techniques. We show that the new vector, pLV-U6-CMV-EGFP-Puro, not only retains the basic features of the original vector but also possesses the function of protein expression. Using this dual expression vector, the stable cell lines expressing transcription factor IIA γ (TFIIAγ) shRNA and WT HA-TFIIAγ or its TFIIAγ mutants were generated and the effects of *TFIIAγ* gene mutations on transcription and protein–DNA interaction were examined. We show that the transfection of the vector expressing TFIIAγ shRNA and *HA-TFIIAγ* fusion gene was able to silence expression of endogenous *TFIIAγ* gene but not affect that of exogenous *HA-TFIIAγ* fusion gene in either transiently transfected cells or stable cell lines. Mutations in the conservative domain between AA_62_ and AA_69_ in TFIIAγ inhibit the activities of AdML core promoter and a few natural promoters. The stable cell line expressing the TFIIAγ N63A mutant reduces expression of endogenous gene compared with the cell line expressing WT TFIIAγ. Protein-DNA binding assays revealed that *TFIIAγ* gene mutations inhibit TFIIAγ binding to promoter DNA. ChIP-qPCR data suggest that the TFIIA N63A mutant inhibits insulin-like growth factor 2 (IGF2) transcription by reducing the recruitments of TFIIAγ, polymerase II (Pol II), TATA box-binding protein (TBP), and TBP associated factor 1 (250 kDa) (TAF1) at its promoter.

## Materials and methods

### Plasmids and reagents

The shRNA-expressing lentiviral vector, pLV-U6-EGFP-Puro, was purchased from Inovogen Tech Co. (China). The pET30a (+) plasmid was obtained from Novagen; and human TFIIAγ subunit and TBP cDNAs were respectively cloned between *BamH*I and *Hind*III in the vector. Restriction enzymes for gene cloning were purchased from New England Biolab. DNA and RNA miniprep kits were obtained from Axygen. PCR and qPCR reagents were from Thermo Scientific. All general chemicals were purchased from the SinoPharm Chemical Reagent Co. (China).

### Site-directed mutagenesis and gene cloning

PCR site-directed mutagenesis for *Xho*I and *Xba*I site mutations was performed using the pLV-U6-EGFP-Puro plasmid as DNA templates. Briefly, 200 ng of the plasmid, 1 μl of 25 μM forward primer, 1 μl of 25 μM reverse primer and 12 μl of ddH_2_O were mixed with 15 μl of PCR Mastermix (2×, Promega) in a 200-μl microtube. PCR was performed in a thermal cycler (GeneAtlas) at the condition of 98°C 20 s, 55°C 10 s and 72°C 5 min for 35 cycles; followed by *Dpn*I digestion and transformation with *E. coli* (DH5a). Positive clones were screened by digestion with *Xho*I and *Xba*I and verified by DNA sequencing (Tsingke Co. China). To perform gene cloning, the DNA fragments containing nine restriction enzyme sites were synthesized and replaced the multiple cloning sites (MCS) in the mutated plasmid. Positive plasmids were screened by digestion with *BamH*I and *EcoR*I enzymes. Next, a CMV promoter was inserted between *Not*I and *Xho*I sites in the plasmid containing a new MCS (see diagram in Figure 2D). The plasmids containing a CMV promoter (pLV-U6-CMV-EGFP-Puro) were screened by digestion with *Not*I and *Xho*I and verified by DNA sequencing. The pLV-U6-CMV-EGFP-Puro vector was subsequently used for cloning of cDNAs, including the DNAs encoding β-tubulin shRNA, HA-β-tubulin, TFIIAγ shRNA, and HA-TFIIAγ.

### Cell culture, transfection, and Western blot

Transformed HEK293 (293T) cells were cultured in a high-glucose DMEM (Hyclone Co.) with supplement of 10% FBS (AusGenex) and 1× penicillin/streptmycin (Thermo Scientific). The 293T cells were seeded in a 12-well plate prior to transfection. After 24 h culture medium was replaced with Opti-MEM (Thermo Scientific), the cells were transfected using 2 μl Turbofect transfection reagent (Thermo Scientific) and 1 μg lentiviral vector expressing β-tubulin shRNA or HA-β-tubulin. Cell samples were harvested at 48 h after transfection, 10 μg of protein from the cell lysate was used for Western blot analysis. Western blot was performed using the antibodies against HA (Sigma-Aldrich, Cat. No. H6908, 1:1000) or against β-tubulin (Sigma-Aldrich, Cat. No. T8328, 1:1000).

### Generation of stable cell lines and luciferase assay

The cDNAs encoding β-tubulin shRNA or TFIIAγ shRNA and the cDNAs encoding HA-β-tubulin or HA-TFIIA were cloned respectively downstream of the U6 promoter and downstream of the CMV promoter in the pLV-U6-CMV-EGFP-Puro plasmid. To obtain human TFIIAγ mutants, PCR site-directed mutagenesis was performed using the vector expressing both TFIIAγ shRNA and HA-TFIIA as DNA templates and the primers containing point mutations at the 63rd, 65th, 67th and 69th amino acids in TFIIAγ, and positive plasmids were screened by DNA sequencing. To generate stable cell lines, the vectors expressing both shRNA and exogenous protein or either of them and the lentiviral packaging vectors, PH1 and PH2 (Innovogen Tech Co.), were co-transfected into 293T cells cultured in a 12-well plate; after 48 h, stable cell lines were selected using puromycin with final concentration of 5 μg/ml and 96-well plates. The expression of endogenous and exogenous protein was determined using Western blot and anti-TFIIAγ antibody (Bolster Biol. Tech. China). To perform luciferase assays, 293T stable cell lines were seeded in 12-well plates and were transfected with the vector expressing a report gene driven by the promoter of AdML or by one of natural promoters, including early growth response protein 1 (EGR1), insulin-like growth factor 2 (IGFII), vascular endothelial growth factor A (VEGFA), amphiregulin (AREG), podcalyxin-like (PODXL), and B-cell lymphoma 2 (BCL2). After 48 h, transiently transfected cells were harvested and then lyzed using 50 μl of lysis buffer from luciferase detection kit (Promega); 3 μl of cell lysate was used for luciferase assay. The assays for each sample were performed in triplicates, and the data obtained were subjected to statistical analysis.

### Recombinant protein preparation and immobilized protein-DNA binding assays

The pET30 (+) plasmids expressing his-tagged human TBP, TFIIAγ, and TFIIAγ mutants were transformed into *E. coli* (BL21 DE3) competent cells. A single colony was picked from a LB plate and inoculated with 3 ml LB medium, bacteria were cultured in a 37°C shaking incubator overnight. After 18 h, 3 ml of bacterial suspension was added into 100 ml LB medium and continually cultured in a 37°C shaking incubator until the value of OD_600_ reached to 0.1. Next, 100 μl of 1M IPTG solution was added into the bacterial cultures to induce protein expression. Bacteria were harvested when the OD_600_ value reached to 0.6, and used for recombinant protein purification according to the manufacturer’s protocol (Qiagen). The concentrations of recombinant proteins were determined using BCA protein assay kit (Thermo Scientific) and the effect of purification was analyzed by Coommasie staining.

Immobilized protein-DNA binding assays were performed using recombinant proteins and biotin-modified AdML core promoter DNA. Briefly, the biotin-modified AdML core promoter DNA (60 bp) was synthesized by Tzingke Biotech Co. (Wuhan, China). Two micrograms of the AdML promoter DNA was mixed with 50 μl of Dynal streptavidin magnetic beads (Thermo Scientific), and the resulting mixture was subsequently incubated overnight on a 4°C rocker. DNA-immobilized beads were precipitated, washed with 1× PBS and suspended with 30 μl binding buffer (10 mM HEPES, 55 mM KCl, 10% glycerol, 0.5 mM EDTA, 1 mM DTT, 0.2 mM PMSF, 0.2 μg/μl BSA, 0.05 μg/μl polydGdC). Next, 1 μl of recombinant TBP and 1 μl of WT TFIIAγ or its mutants were mixed with 30 μl of the DNA-immobilized beads, and the reaction mixture was incubated at 30°C for 1 h on a rotating rocker. After incubation, protein-binding beads was washed for four times using 1× PBS, 30 μl of SDS-loading buffer was added to the beads and boiled for 10 min in a 100 °C heat block. DNA-binding protein was obtained by centrifugation and detected by Western blot.

### RT-qPCR and ChIP assay

293T stable cell lines expressing TFIIAγ shRNA and WT TFIIAγ or its N63A mutant were cultured in 6-well plates. At 80% confluence, stable cell lines were harvested, total RNA was extracted from the cell lines using RNA miniprep kit (Axygen); cDNA was synthesized using 1 μg of total RNA and two units of reverse transcriptase according to the manufacturer’s manual (Thermo Scientific). 0.5 μl of reaction mixture was used for qPCR, qPCR was performed using SYBR green reagent (Roche) and a real-time PCR detection system (Bio-Rad). Quantitative PCR data were analyzed with CFX Manager 3.1 software (Bio-Rad).

To perform ChIP assays, 293T stable cell lines expressing TFIIAγ shRNA and WT TFIIAγ or its N63A mutant were cultured in 10-cm dishes. At 85% confluence, stable cell lines were fixed, harvested, and lysed with ChIP lysis buffer (1% SDS, 10 mM EDTA, 50 mM Tris-HCl, pH 8). ChIP assays were performed as described previously [27]. Quantitative PCR for ChIP samples was performed using SYBR Green reagent (Roche) and a real-time PCR detection system (Bio-Rad). The relative occupancy was obtained by comparing the enrichment of promoter DNA from the ChIP sample with that from the input; the input DNA used for qPCR was equivalent to 0.01% of the original sample used for the ChIP assay. The antibodies used for ChIP assays were purchased from Santa Cruz Biotech (Pol II: SC-21751, TBP: SC-204, transcription factor IIB [TFIIB]: SC-225, TAF1: SC-735, TBP associated factor 4 [135 kDa] [TAF4]: SC-136093) except that TFIIAα/β antibody was from FrdBio. Co. (Wuhan, China) and HA antibody was from Sigma-Aldrich (Cat. No. H6908).

## Results

### Construction of a lentiviral vector expressing both RNA and protein

**Figure 1:**
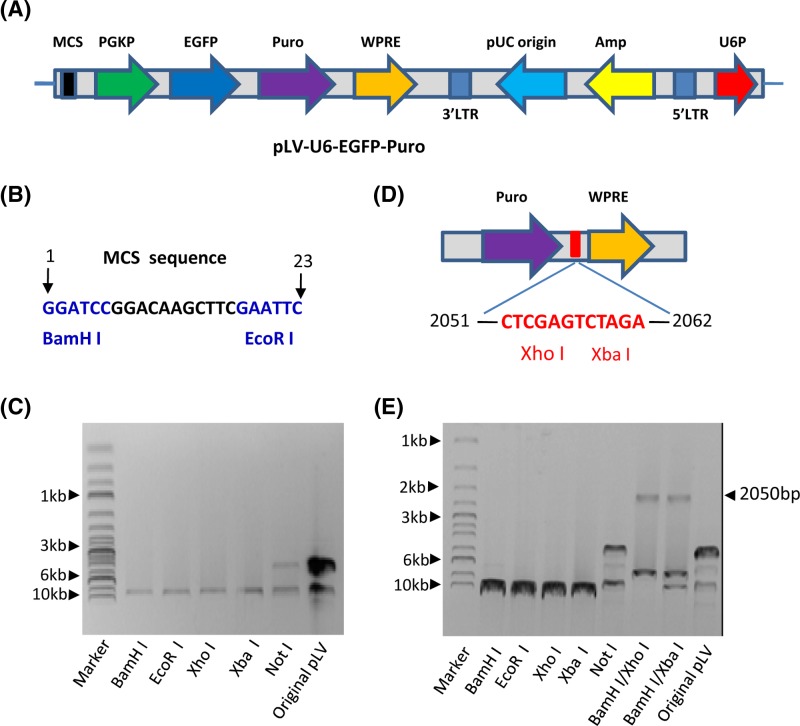
Characterization of the pLV-U6-EGFP-Puro lentiviral vector (**A**) A scheme showing basic elements included in the pLV-U6-EGFP-Puro plasmid. (**B**) The sequence and location of MCS in the pLV-U6-EGFP-Puro plasmid. The MCS sequence contains only two restriction enzyme sites, *BamH*I and *EcoR*I (blue). (**C**) An agarose gel image showing the result of enzyme digestion of the pLV-U6-EGFP-Puro plasmid. The pLV-U6-EGFP-Puro plasmid was digested with the enzymes as indicated in the Figure, followed by detecting with agarose gel electrophoresis and imaging with ChemiDoc MP imaging system (Bio-Rad). (**D**) A scheme showing the positions of two restriction enzyme sites, *Xho*I and *Xba*I, in the pLV-U6-EGFP-puro plasmid. DNA-sequencing was performed using the pLV-U6-EGFP-Puro plasmid, and the restriction enzyme sites included in the vector were analyzed. (**E**) Restriction enzyme digestion confirmed that the pLV-U6-EGFP-Puro plasmid contains *Xho*I and *Xba*I sites between 2051 and 2063 bp downstream of puromycin resistant gene (Puro). Plasmid DNA was digested using the enzymes as indicated in the Figure; the image for agarose gel electrophoresis was obtained as described in (C). Abbreviation: original pLV, pLV-U6-EGFP-Puro plasmid.

Conventional methods to study the effects of gene mutations at the cellular level have prominent drawbacks due to the interference of endogenous gene expression; we supposed that this disadvantage could be overcome by using a dual expression lentiviral vector. To achieve this goal, a shRNA-expressing lentiviral vector, pLV-U6-EGFP-Puro, was used to construct a dual expression lentiviral vector (Figure 1A). Since the pLV-U6-EGFP-Puro plasmid contains only two restriction enzyme sites, *BamH*I and *EcoR*I, at the MCS (Figure 1B), the limited enzyme sites obstruct the construction of the dual expression lentiviral vector. To solve this problem, DNA-sequencing for the pLV-U6-EGFP-Puro plasmid was performed, and restriction enzyme sites including the vector were analyzed with online software (Webcutter 2.0). The result revealed that the original vector does not contain a number of common restriction enzyme sites, including *Not*I, *PMe*I, *Hpa*I, *Bcl*I, and *Sma*I, but contains only one site for *BamH*I, *EcoR*I, *Xho*I, and *Xba*I (data not shown). To confirm this result, the pLV-U6-EGFP-Puro plasmids were digested with restriction enzymes and detected by agarose gel electrophoresis, the result from agarose gel electrophoresis showed that the plasmid was able to be linearized by the enzymes predicted by software analysis (Figure 1C). In addition, two restriction enzyme sites, including *Xho*I and *Xba*I, are located between the puromycin-resistant gene and the WPRE element based on software analysis result (Figure 1D). To verify the position of these two sites, the pLV-U6-EGFP-Puro plasmid was digested with *BamH*I and *Xho* I or with *BamH* I and *Xba* I, and detected by agarose gel electrophoresis. As expected, around 2050 bp fragment has been identified from the plasmid (Figure 1E). Since these two sites are not included in the key elements of this lentiviral vector, it suggests that mutations of both *Xho*I and *Xba*I sites might not affect the function of the vector and that these two enzyme sites along with those not included in the pLV-U6-EGFP-Puro plasmid can be utilized to construct a dual expression vector.

**Figure 2:**
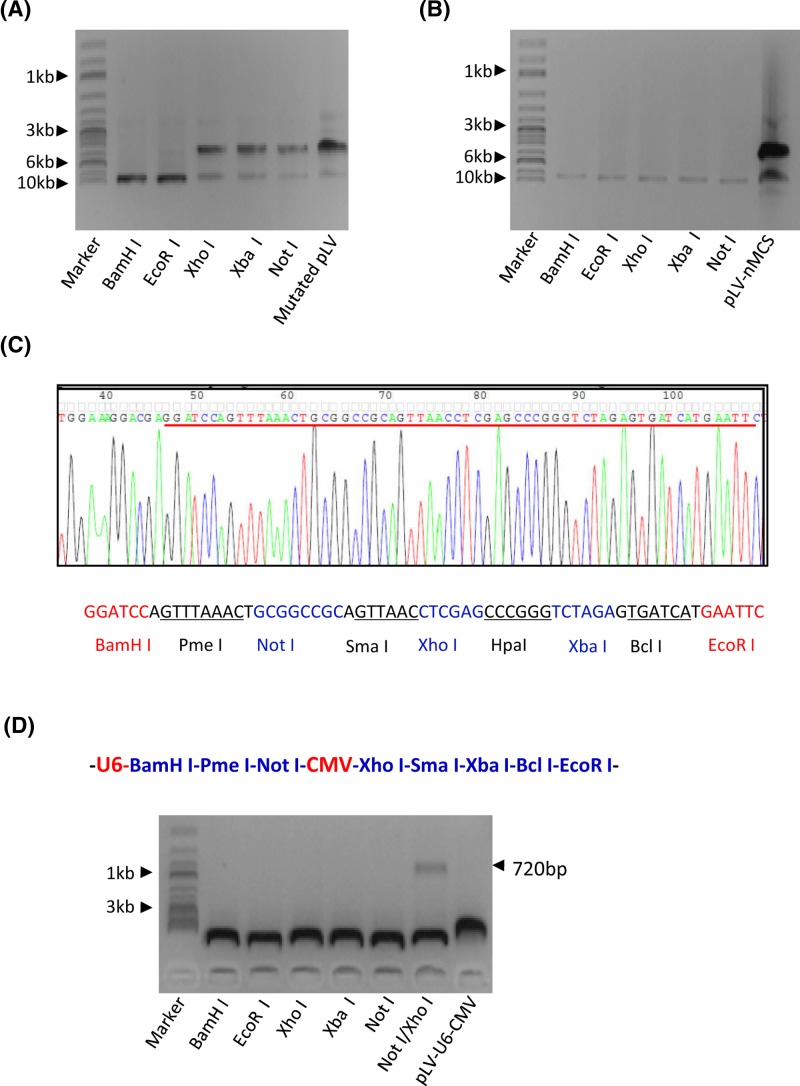
Construction of a novel dual expression lentiviral vector (**A**) Analysis of restriction enzyme digestion for the pLV-U6-EGFP-Puro plasmid that contains mutated *Xho*I and *Xba*I sites. PCR site-directed mutagenesis was performed using the pLV-U6-EGFP-Puro plasmid and the primers containing mutated *Xho*I and *Xba*I sites. Positive plasmids were screened by enzyme digestion and analyzed by agarose gel electrophoresis and then imaged as for Figure 1C. (**B**) The DNA fragment containing nine restriction sites was cloned into the pLV-U6-EGFP-Puro plasmid. A DNA fragment containing nine restriction sites was synthesized and cloned between *BamH*I and *EcoR*I sites in the pLV-U6-EGFP-Puro plasmid. Positive plasmids were screened by enzyme digestion; the gel image was obtained as for Figure 1C. (**C**) DNA sequencing result for the new MCS DNA fragment containing nine restriction enzymes. (**D**) The CMV promoter was cloned into the plasmid containing the new MCS. Positive plasmids were screened and analyzed as described in Figure 1C. Abbrreviations: mutated pLV, pLV-U6-EGFP-Puro plasmid with mutated *Xho*I and *Xba*I sites; pLV-nMCS, pLV-U6-EGFP-Puro plasmid with a new MCS DNA fragment.

To construct a dual expression lentiviral vector, both *Xho*I and *Xba*I sites in the pLV-U6-EGFP-Puro plasmid were initially mutated using PCR site-directed mutagenesis, mutants were screened through digestion with *Xho*I and *Xba*I enzymes and verified by DNA sequencing. The image from agarose gel electrophoresis showed that the mutated plasmid was resistant to *Xho*I and *Xba*I enzymes (Figure 2A). Next, the DNA fragments containing nine restriction enzyme sites were synthesized and replaced the MCS in the pLV-U6-EGFP-Puro plasmid. The data demonstrated that the resulting plasmid was able to be linearized by *Xho*I, *Xba*I, and *Not*I enzymes (Figure 2B). DNA sequencing confirmed that the DNA fragment with nine restriction sites was successfully cloned into the pLV-U6-EGFP-Puro plasmid (Figure 2C). To obtain a dual expression vector, the CMV promoter DNA was amplified from the pcDNA 3.1(+) plasmid by PCR and cloned between *Not*I and *Xho*I sites in the vector containing a new MCS fragment. As shown in Figure 2D, a 720 bp DNA fragment has been identified from the positive clone after enzyme digestion, indicating the dual expression vector containing U6 and CMV promoters has been constructed, and this vector is thereafter designated as pLV-U6-CMV-EGFP-Puro.

### Functional validation of the pLV-U6-CMV-EGFP-Puro vector

**Figure 3:**
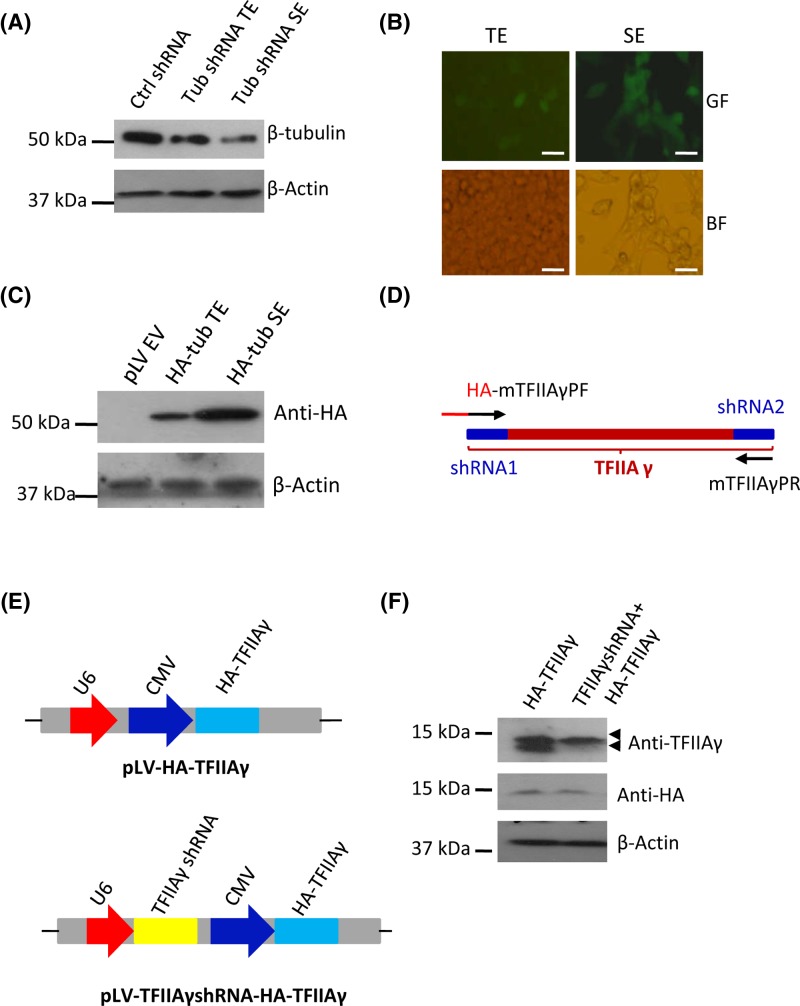
Functional validation of the pLV-U6-CMV-EGFP-Puro plasmid (**A**) The pLV-U6-CMV-EGFP-Puro vector can express shRNA. The pLV-U6-CMV-EGFP-Puro plasmid expressing β-tubulin shRNA was transfected into 293T cells in the presence or absence of packaging vectors. β-tubulin expression was analyzed using both transiently transfected cells and stable cell lines and Western blot. (**B**) Fluorescence microscopy for the cells that transiently or stably express β-tubulin shRNA. The cells obtained in (A) were imaged under fluorescence microscope. Scale bar in each image represents 50 μm. (**C**) The pLV-U6-CMV-EGFP-Puro plasmid can express protein. The pLV-U6-CMV-EGFP-Puro plasmid expressing HA-β-tubulin was transfected into 293T cells in the presence or absence of packaging vectors, HA-β-tubulin expression was analyzed using transiently transfected cells and stable cell line and Western blot. (**D**) A diagram showing TFIIAγ cDNA regions used for TFIIAγ shRNA design and the primers for modification of TFIIAγ cDNA. HA DNA fragment was added to the front of TFIIAγ forward primer to form the HA- mTFIIAγPF fusion primer, the third base of each codon in mTFIIAγPF and mTFIIAγPR was mutated. (**E**) A diagram showing the plasmids expressing HA-TFIIAγ only (pLV-HA-TFIIAγ) or expressing both TFIIAγ shRNA and HA-TFIIAγ (pLV-TFIIAγshRNA-HA-TFIIAγ). (**F**) The pLV-TFIIAγshRNA-HA-TFIIAγ plasmid can express TFIIAγ shRNA and HA-TFIIAγ simultaneously. The pLV-TFIIAγshRNA-HA-TFIIAγ and pLV-HA-TFIIAγ plasmids were respectively transfected into 293T cells; after 48 h, expression of TFIIAγ and HA-TFIIAγ was analyzed using transiently transfected cells and Western blot. Abbreviations: BF, bright field; GF, green fluorescence; SE, stable expression; TE, transient expression.

Considering that the pLV-U6-CMV-EGFP-Puro plasmid was constructed from the pLV-U6-EGFP-Puro plasmid, and the pLV-U6-EGFP-Puro plasmid has gone through a number of alterations, including gene mutations, DNA fragment insertion and promoter cloning; the basic functions of the dual expression lentiviral vector have to be validated. To achieve this goal, three different DNA fragments that encode β-tubulin shRNA molecules were cloned downstream of the U6 promoter in the dual expression vector. The β-tubulin shRNA-expressing plasmids were transfected into 293T cells in the presence or absence of the lentiviral packaging vectors. After 48 h, transiently transfected cells were harvested and protein expression was detected by Western blot. In parallel experiments, stable cell lines were screened with puromycin and 96-well plates and analyzed along with transiently transfected cells. Figure 3A shows that β-tubulin expression was reduced in the cells expressing β-tubulin shRNA compared with the cells expressing control shRNA, indicating that the pLV-U6-CMV-EGFP-Puro vector can effectively express shRNA to silence endogenous gene expression. Next, both transiently transfected cells and stable cell lines were observed under a fluorescent microscope. The result showed that both types of cells were able to express green fluorescent protein (Figure 3B); indicating that EGFP selection marker in the pLV-U6-CMV-EGFP-Puro plasmid is functional. In addition, the stable cell line expressing β-tubulin shRNA has been obtained through puromycin selection, and was able to express EGFP after a number of passages (Figure 3B), suggesting that the vector has been integrated into the genome of 293T cells and that puromycin selection marker and genome integration element remain functional. To determine whether the pLV-U6-CMV-EGFP-Puro plasmid could express a protein, a *HA-β-tubulin* fusion gene was cloned downstream of the CMV promoter, the vector expressing the HA-β-tubulin fusion protein was transfected into 293T cells in the presence or absence of packaging vectors. Transiently transfected cells and stable cell lines were harvested to analyze HA-β-tubulin expression. Western blot showed that both types of cells were able to express HA-β-tubulin protein (Figure 3C), indicating that the pLV-U6-CMV-EGFP-Puro vector can be utilized to express exogenous proteins. Taken together, these data indicate that the pLV-U6-CMV-EGFP-Puro plasmid not only retains the basic functions of the original vector but also gains the function of protein expression.

### The pLV-U6-CMV-EGFP-Puro plasmid can concurrently express both shRNA and protein

Since the pLV-U6-CMV-EGFP-Puro plasmid contains both U6 and CMV promoters, we next determined whether shRNA and protein could be expressed simultaneously using the dual expression vector. To this end, the cDNAs that encode TFIIAγ shRNA or HA-TFIIAγ fusion protein were cloned respectively downstream of the U6 promoter and downstream of the CMV promoter. In order to prevent HA-TFIIAγ mRNA from degradation by TFIIAγ shRNA, the TFIIAγ cDNA regions that were used to design TFIIA shRNA were mutated at the third base of each codon (Figure 3D). The vectors expressing HA-TFIIAγ only or both TFIIAγ shRNA and HA-TFIIAγ (Figure 3E) were respectively transfected into 293T cells; the cell lysate from transiently transfected cells was used to analyze protein expression by Western blot. As shown in Figure 3F, endogenous TFIIAγ expression was reduced by transfection of the vector expressing TFIIAγ shRNA and HA-TFIIAγ compared with that by transfection of the vector expressing HA-TFIIAγ only, and the cells transfected with either of the vectors were able to express HA-TFIIAγ protein; indicating that endogenous *TFIIAγ* gene expression was silenced by TFIIAγ shRNA; whereas, exogenous *HA-TFIIAγ* fusion gene expression was not affected. These results suggest that the pLV-U6-CMV-EGFP-Puro plasmid can be used to express both shRNA and protein concurrently and to investigate the effects of gene mutations at the cellular level.

### TFIIAγ mutations can inhibit the activities of promoters and endogenous gene expression

**Figure 4:**
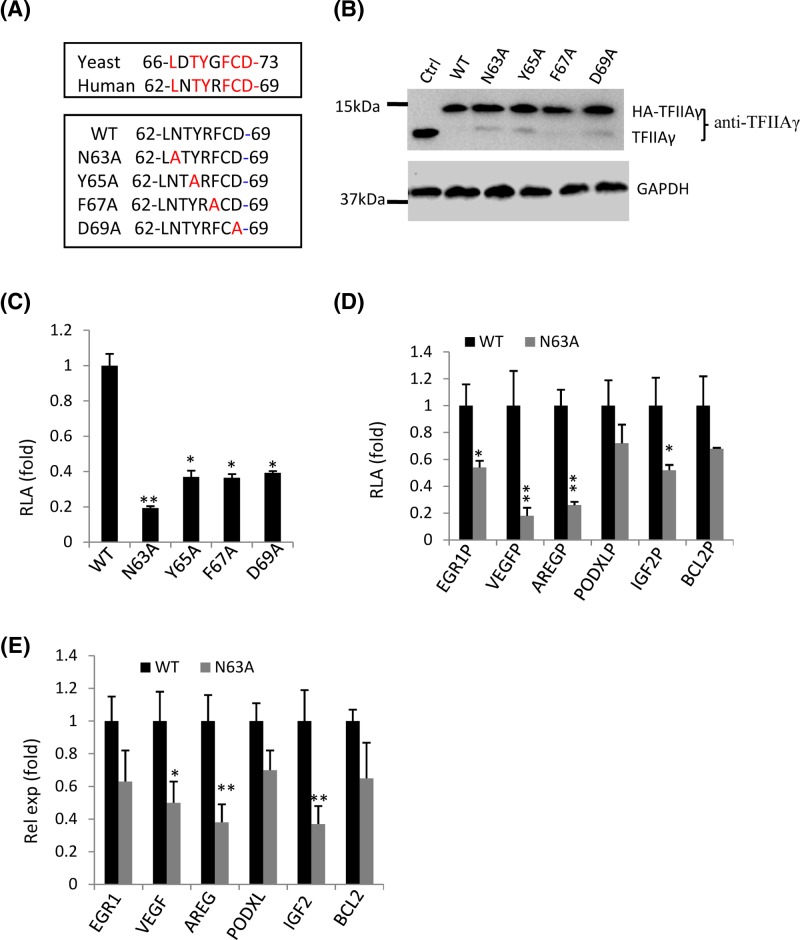
Human *TFIIAγ* gene mutations inhibited promoter activity and endogenous gene expression (**A**) A diagram showing the conservative amino acids between yeast TOA2 and human TFIIAγ (top panel) and the mutation sites within human TFIIAγ (bottom panel). (**B**) Western blot analysis for 293T stable cell lines expressing TFIIAγ shRNA and WT HA-TFIIAγ or its mutants. 293T cells were transfected with the plasmids expressing TFIIAγ shRNA and WT HA-TFIIAγ or its derivatives in the presence of the lentiviral packaging vectors. Expression of TFIIAγ and HA-TFIIAγ was analyzed by Western blot. (**C**) Mutations of *TFIIAγ* gene significantly inhibited the activity of AdML promoter. 293T stable cell lines were transfected with the AdML promoter-driving reporter gene vectors; after 48 h, the cells were harvested for luciferase assays. (**D**) The effect of the N63A mutant on the activities of natural promoters. 293T stable cell lines, including WT and N63A, were transfected using the vectors expressing a reporter gene that is driven by one of the natural promoters as indicated; after 48 h, the cells were harvested for luciferase assays. (**E**) RT-qPCR result showing the effect of the N63A mutant on expression of endogenous genes. Total RNA was extracted from stable cell lines, including WT and N63A, the cDNA for each sample was synthesized and detected by quantitative PCR. Each *column* in (C–E) represents the mean ± S.D. of three independent experiments. *, *P*<0.05; **, *P*<0.01; *P*-values were obtained with one way ANOVA. Abbreviation: RLA, relative luciferase activity

General TFIIA plays an important role in RNA polymerase II-mediated transcriptional initiation and acts as a co-activator or an anti-repressor to regulate transcription activity [28]. Human TFIIA consists of three subunits, α, β, and γ; α and β subunits are encoded by a gene; whereas, γ subunit is encoded by another gene [28]. In yeast, TFIIA comprises two subunits, TOA1 and TOA2; TOA1 is the counterpart of human TFIIAα/β, TOA2 is equivalent to human TFIIAγ [28]. Previous studies have shown that yeast TOA2 interacts with TBP in the TFIIA-TBP-DNA complex [29,30]. In addition, human TFIIAγ Y65A mutant can inhibit gene transcription [31]. Protein sequence analysis showed that the amino acids between AA_66_ and AA_73_ in TOA2 are conservative in human TFIIAγ (Figure 4A); moreover, the 67th, 69th, 71th and 73th amino acids in yeast TOA2 were identified to interact with TBP in the TFIIA-TBP-DNA complex [29,30]. Thus, we supposed that mutations of the counterparts in human TFIIAγ (Figure 4A) could also affect gene transcription in human cells. To confirm this hypothesis, the dual expression vector obtained above was used to investigate the effects of *TFIIAγ* gene mutations. Four TFIIAγ mutants were generated using the pLV-TFIIAγ shRNA-HA-TFIIAγ vector as DNA templates (Figure 4A). The vectors expressing TFIIAγ shRNA and WT HA-TFIIAγ or its mutants were respectively transfected into 293T cells in the presence of the lentiviral packaging vectors. Stable cell lines were obtained by screening with puromycin and 96-well plates, TFIIAγ and HA-TFIIAγ expression in these cell lines was subsequently detected by Western blot. Figure 4B shows that endogenous TFIIAγ expression was reduced in the cell lines expressing TFIIAγ shRNA and WT HA-TFIIAγ (or its mutants) compared with that in control cell line; whereas, exogenous WT HA-TFIIAγ and its mutants were expressed in all of the cell lines except the control cell line, indicating that the cell lines with depletion of endogenous TFIIAγ and expression of exogenous HA-TFIIAγ or its mutants were achieved.

To determine the effects of *TFIIAγ* gene mutations on transcription, the stable cell lines obtained above were transfected with the AdML-driving reporter gene vector, the cell lysate from transiently transfected cells were used to detect luciferase activity. As shown Figure 4C, all TFIIAγ mutants significantly inhibited the activity of the AdML promoter compared with WT TFIIAγ, although the N63A mutant showed greater inhibition to transcription than the other three mutants. To test whether *TFIIAγ* gene mutations affected the activities of other promoters, a number of natural promoters were cloned into pGL3-basic reporter vector. Given that the N63A mutant showed more severe inhibition to the AdML promoter activity than other mutants, the vectors that express a reporter gene driven by different natural promoters were respectively transfected into two stable cell lines, WT and N63A; and luciferase assays were performed using the cell lysate from transiently transfected cells. The data showed that the N63A mutant significantly inhibited the activity for most of the promoters tested in the assays compared with WT TFIIAγ; whereas, the activities of BCL2 and PODXL promoters were not much affected (Figure 4D). To verify this observation, total RNA was extracted from two stable cell lines (WT and N63A); and endogenous gene expression was analyzed by RT-qPCR. Figure 4E illustrates that the N63A mutant significantly inhibited expression of *AREG, IGF2*, and *VEGF* genes but did not severely affect the expression of *EGR1, PODXL*, and *BCL2* genes. Taken together, mutations in the conservative domain between AA_62_ and AA_69_ in human TFIIAγ can inhibit the activities of promoters and endogenous gene expression.

### Mutations of *TFIIAγ* gene inhibit TFIIAγ protein binding to the promoters *in vitro* and *in vivo*

**Figure 5:**
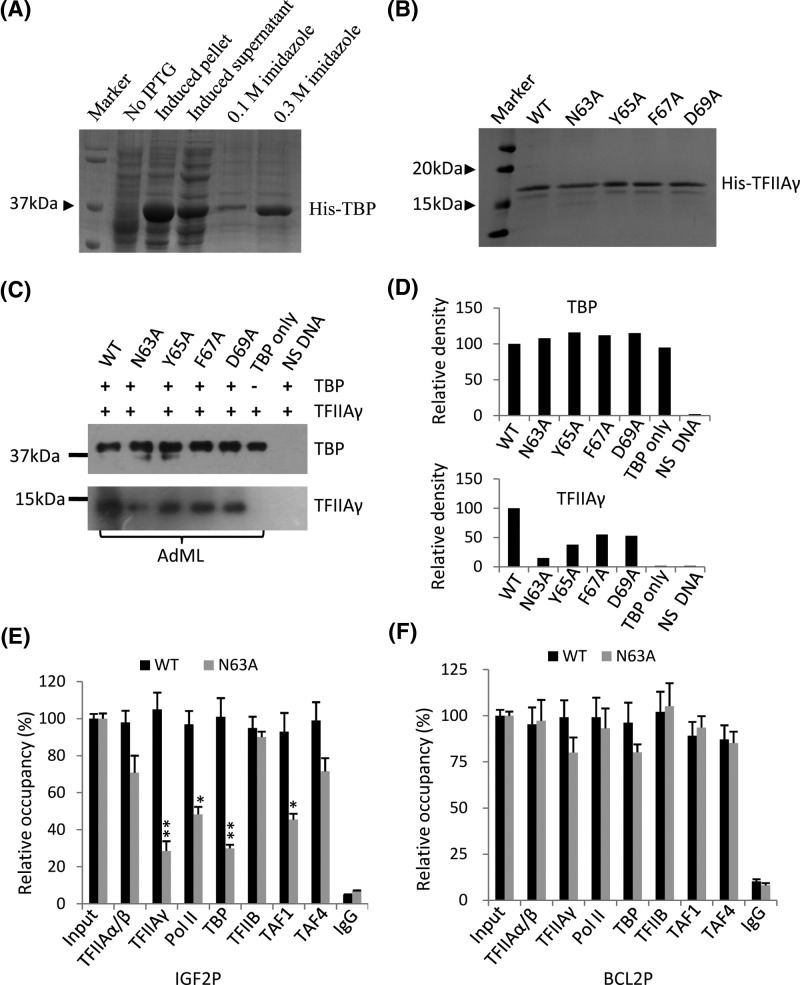
Mutations of *TFIIAγ* gene repressed TFIIAγ binding to the TATA-containing promoters (**A**) Coomasie staining showing the effect of expression and purification for human recombinant TBP. Recombinant TBP was expressed in *E. Coli* BL21 (DE3), purified using nickel agarose and detected by SDS–PAGE and Coomasie staining. (**B**) Coomasie staining showing the effect of purification for recombinant TFIIAγ and its mutants. Recombinant WT TFIIAγ and its mutants were prepared and detected as described in (A). (**C, D**) Mutations of TFIIAγ gene reduced TFIIAγ binding to the AdML promoter *in vitro*. Immobilized DNA-protein binding assays were performed using the biotin-modified AdML core promoter and recombinant proteins TBP and WT TFIIAγ or its mutants. The DNA-binding protein was detected by Western blot and the antibodies against TBP or TFIIAγ (C). Density of the bands in (C) was quantified and shown in (D). (**E**) ChIP-qPCR showing the effect of the N63A mutant on the occupancies of components of the Pol II transcription machinery at the IGF2 promoter. (**F**) ChIP-qPCR showing the effect of the N63A mutant on the occupancies of components of the Pol II transcription machinery at the IGF2 promoter. Each *column* in (E) and (F) represent the mean ± S.E.M. of three independent experiments.*, *P*<0.05; **, *P*<0.01; *P*-values were obtained with one way ANOVA.

Previous study has shown that human TFIIAγ Y65A mutant inhibits formation of the TFIIA-TBP-DNA complex [30]; moreover, mutations of *TFIIAγ* gene repressed the activities of promoters and endogenous gene expression (Figure 4C–E). We next tested if *TFIIAγ* gene mutations inhibited promoter activity through affecting TFIIAγ binding to AdML promoter. To this end, recombinant TBP and WT TFIIAγ or its mutants were expressed in *E. coli* BL21(DE3) and purified with Ni-NTA agarose, the proteins purified were analyzed by Coommasie staining. As shown in Figure 5A,B, these recombinant proteins have been expressed in bacteria and purified successfully. Protein-DNA binding assays were performed using recombinant TBP, TFIIAγ, and the AdML core promoter DNA. Western blot showed that TFIIAγ protein mutants inhibited their binding to the AdML core promoter compared with WT TFIIAγ, although TBP binding to the AdML promoter was not affected by the mutants (Figure 5C,D). Furthermore, the N63A mutant showed greater inhibition to its binding to DNA than other TFIIAγ mutants; this result is consistent with that obtained in reporter gene assays (Figure 4C), suggesting that the transcriptional inhibition mediated by *TFIIAγ* gene mutations could be caused by the reduced binding of TFIIAγ to the promoters.

Considering that the IGF2 promoter contains a canonical TATA box and that IGF2 expression was severely affected by the N63A mutant; in contrast, BCL2 promoter does not contain the TATA box and its expression was not affected by the N63A mutant (Figure 4E). Next, we asked how *TFIIA gene* mutations affected transcription of *igf2* and *bcl2* genes. To address this question, ChIP assays for RNA Pol II and several general transcription factors and cofactors were performed using the stable cell lines expressing both TFIIAγ shRNA, and WT HA-TFIIAγ or its N63A mutant. ChIP-qPCR data showed that the N63A mutant significantly reduced the occupancies of TFIIAγ, Pol II, TBP, and TAF1 at the IGF2 promoter compared WT TFIIAγ. Unexpectedly, the occupancies of TFIIAα/β, TFIIB, and TAF4 at this promoter were not significantly affected by the N63a mutant (Figure 5E). These results suggest that the N63A mutant inhibits IGF2 transcription by reducing the recruitments of TFIIAγ, Pol II, TBP, and TAF1 at its promoter. In addition, the occupancies for components of the Pol II transcription machinery at the BCL2 promoter between WT TFIIAγ and the N63A mutant did not show significant difference (Figure 5F). Taken together, these data suggest that the N63A mutant can influence the recruitments of Pol II and general transcription factor at the TATA box-containing promoters but not at the TATA box-less promoters.

## Discussion

In the present study, we constructed a novel lentiviral vector expressing both shRNA and proteins using the shRNA-expressing lentivral vector (pLV-U6-EGFP-Puro) and combined molecular techniques. We show that the new vector, pLV-U6-CMV-EGFP-Puro, not only retains the basic features of the original vector but also gains function of protein expression (Figure 3A–C); and that the dual expression vector can be used to silence endogenous TFIIAγ and express exogenous HA-TFIIAγ proteins (Figure 3E,F). Previous study has shown that dual expression lentiviral vectors can be generated using Invitrogen Gateway system; however, the recombined vectors were used to only test protein depletion by siRNA or protein overexpression [32]. Gillanders’ research group has constructed several dual expression vectors based on siRNA expression vector (pSicoR) and confirmed that the dual expression vector can concurrently express shRNA and GFP-tagged proteins [26]. However, the effects of gene mutations have not been investigated using a dual expression vector in the previous studies. In the present study, we have successfully applied the novel dual expression vector to investigating the effects of gene mutations at the cellular level; thus, the purpose of our study is distinct from those of previous studies. The method to study the effects of gene mutations using the dual expression vector has great advantages over the previous methods in which the experiments for protein depletion and expression rescue were performed separately [21,22], because our novel method costs less labour, time, and funds. In addition, our dual expression vector contains a number of restriction enzyme sites (Figure 2D); and the target gene or shRNA can be replaced with other cDNAs encoding small RNA or proteins. Therefore, our dual expression vector can be applied to other research purposes such as expression of guided RNA and CRIPSR-Cas9 protein.

We demonstrate that the mutations in the conservative domain between AA_62_ and AA_69_ in TFIIAγ inhibited the activities of several promoters and endogenous gene expression (Figure 4). Reporter assays showed that the TFIIAγ Y65A mutant showed significant inhibition to AdML promoter activity. This result is consistent with that from previous study [31]; however, the N63A mutant more severely inhibited the activity of AdML promoter than other mutants, including the Y65A mutant (Figure 4C). The studies on the TFIIA-TBP-DNA ternary structure suggest that the conservative domain between AA_62_ and AA_69_ in human TFIIAγ might associate with AdML promoter through interacting with TBP[29,30]. We confirmed that the mutations of the conservative domain of human TFIIAγ, indeed repressed TFIIAγ binding to the AdML core promoter (Figure 5C), suggesting that the interaction between TFIIAγ and TBP is conservative in both yeast and human. ChIP assays showed that the TFIIAγ N63A mutant significantly reduced the occupancies of TFIIAγ, Pol II, TBP, and TAF1 at the IGF2 promoter (Figure 5E). Since TBP and TAF1 can bind respectively to the TATA box and the initiator elements to regulate gene transcription [28], the N63A mutant likely inhibits IGF2 transcription by reducing TBP, TAF1, and Pol II binding to the IGF2 promoter. In addition, we observed the difference of TBP binding to promoters between *in vitro* and *in vivo*. It is possible that the binding of TBP to promoters could be affected by TBP-associated factors (TAFs) and other factors *in vivo*; whereas, it was mainly determined by the TATA box in an *in vitro* assay. Unexpectedly, the TFIIAγ N63A mutant did not significantly affect TFIIAα/β occupancy at the IGF2 promoter, although both are the subunits of TFIIA. Previous study showed that TFIIAα/β depletion and TFIIAγ depletion can cause different effects on expression of some genes [33]; thus, it is possible that TFIIAα/β and TFIIAγ could function separately in transcriptional regulation *in vivo*. The N63A mutant did not affect transcription of BCL2 gene and the occupancies of components of the Pol III transcription machinery at the BCL2 promoter (Figure 5F). It has been shown that the interaction between TFIIAγ and TBP was identified in the TFIIA-TBP-DNA complex where the promoter DNA (yeast CYC1) contains a canonical TATA box [29,30]; thus, it is reasonable that the N63A mutant only affected the occupancies of Pol II transcription machinery at the TATA-containing promoter (IGF2) but not at the TATA-less promoter (BCL2). Taken together, the conservative domain between AA_62_ and AA_69_ in TFIIAγ can modulate gene transcription and Pol II transcription machinery recruitment at promoters; these results provide novel insights into the regulatory mechanism of gene transcription mediated by RNA polymerase II.

## Conclusion

In the present study, a dual expression lentiviral vector (pLV-U6-CMV-Puro-EGFP) was constructed using a series of DNA manipulating techniques. This vector not only retains basic functions of the original lentiviral vector but also gains the function of protein expression. Transfection experiments confirmed that the vector can be used to express shRNA and protein concurrently. The study on the effects of *TFIIAγ* gene mutations revealed that the conservative domain between AA_62_ and AA_69_ in human TFIIAγ can regulate gene transcription by altering Pol II transcription machinery recruitment at promoters. Collectively, the present study provides a novel method that is used to investigate the effects of gene mutations at the cellular level.

## Abbreviations

AdML: Adenovirus major late promoter
AREG: amphiregulin
BCL2P: B-cell lymphoma 2 promoter
CRISPR: clusters of regularly interspaced short palindromic repeats
EGR1: early growth response protein 1
IGF2P: insulin-like growth factor 2 promoter
IPTG: Isopropyl-beta-D-thiogalactopyranoside
MCS: multiple cloning sites
PMSF: Phenylmethylsulfonyl fluoride
PODXL: podcalyxin-like
Pol II: polymerase II
qPCR: quantitative polymerase chain reaction
shRNA: small hairpin RNA
TAF1: TBP associated factor 1 (250 kDa)
TAF4: TBP associated factor 4 (135 kDa)
TALEN: transcription activator-like effector nuclease
TBP: TATA box-binding protein
TFIIA: transcription factor IIA
TFIIB: transcription factor IIB
VEGF: vascular endothelial growth factor
WPRE: Woodchuck Hepatitis Virus (WHP) Posttranscriptional Regulatory Element
ZFNs: Zinc finger nucleases
